# Associations between modifiable lifestyle factors and multidimensional cognitive health among community-dwelling old adults: stratified by educational level

**DOI:** 10.1017/S1041610217003076

**Published:** 2018-02-15

**Authors:** Manqiong Yuan, Jia Chen, Yaofeng Han, Xingliang Wei, Zirong Ye, Liangwen Zhang, Y. Alicia Hong, Ya Fang

**Affiliations:** 1 State Key Laboratory of Molecular Vaccinology and Molecular Diagnostics, School of Public Health, Xiamen University, Xiamen 361102, China; 2 Key Laboratory of Health Technology Assessment of Fujian Province, School of Public Health, Xiamen University, Xiamen 361102, China; 3 School of Public Health, Texas A&M University, College Station, TX, USA

**Keywords:** multi-domain cognitive health, lifestyle factors, education-specific, the elderly, proportional odds model

## Abstract

**Background:**

Cognition is multidimensional, and each domain plays a unique and crucial part in successful daily life engagement. However, less attention has been paid to multi-domain cognitive health for the elderly, and the role of lifestyle factors in each domain remains unclear.

**Methods:**

We conducted a cross-sectional study of 3,230 older adults aged 60+ years in Xiamen, China, in 2016. The Montreal Cognitive Assessment (MoCA) was used to measure general cognition and six specific sub-domains. To account for educational effects, we adjusted the MoCA score and divided respondents into three education-specific groups (low, moderate, and high education groups with ≤5, 6~8, and ≥9 years of education, respectively). A series of proportional odds models were used to detect the associations between two categories of lifestyle factors – substance abuse (cigarette and alcohol) and leisure activity (TV watching, reading, smartphone use, social activity, and exercise) – and general cognition and the six sub-domains in those three groups.

**Results:**

Among the 3,230 respondents, 2,617 eligible participants were included with a mean age of 69.05 ± 7.07 years. Previous or current smoking/drinking was not associated with MoCA scores in the whole population, but unexpectedly, the ex-smokers in the low education group performed better in general cognition (OR = 2.22) and attention (OR = 2.05) than their never-smoking counterparts. Modest TV watching, reading, and smartphone use also contributed to better cognition among elderly participants in the low education group. For the highly educated elderly, comparatively longer reading (>3.5 hours/week) was inversely associated with general cognition (OR = 0.53), memory (OR = 0.59), and language (OR = 0.54), while adequate exercise (5~7 days/week) was positively related to these factors with OR = 1.48, OR = 1.49, and OR = 1.53, respectively. For the moderately educated elderly, only modest reading was significantly beneficial.

**Conclusions:**

Lifestyle factors play different roles in multidimensional cognitive health in different educational groups, indicating that individual intervention strategies should be designed according to specific educational groups and different cognitive sub-domains.

## Introduction

Population aging is considered one of the most significant social problems around the world today. According to data from the Department of Economic and Social Affairs of the United Nations, 12.3% of people worldwide (900 million) were over the age of 60 years in 2015, and this figure is projected to increase by 16.5% by 2030 (1.40 billion). Alzheimer's disease, as an aging-associated disease, has consistently aroused public awareness worldwide for not only affecting individuals’ quality of life but also imposing substantial financial burdens on society and emotional burdens on caregivers (Callahan, 2017). However, less attention has been paid to cognitive decline *per se*, particularly from a public health perspective. It is reported that numerous age-related changes in cognition are highly relevant to daily activities and have considerable importance to the public (IOM, 2015).

Cognition is multidimensional, encompassing processes such as attention, memory, executive functioning, logic, and language function. Collectively, these different domains of cognition play a fundamental and crucial part in successful engagement in daily life. For example, declines in memory may influence people's adherence to a medication schedule (Insel *et al.*, 2006), deterioration in executive functioning is linked to declines in ability to perform instrumental activities of daily living (Bell-McGinty *et al.*, 2002), and other degenerations in cognitive abilities can increase the risk of making errors in financial decisions (Samanez-Larkin *et al.*, 2012) and damaging performance on technology-based tasks such as searching the internet (Czaja *et al.*, 2013). Hence, maintaining one's cognition in different domains is quite important to assure the quality of one's late life. Furthermore, it is quite necessary to gain a deep understanding of the beneficial and deleterious factors affecting cognition in different domains and to have a prevention guide.

Cognitive decline is considered to be multifactorial, with unmodifiable factors (e.g. age and genetics) and modifiable factors (e.g. environmental factors and lifestyle factors) (Ngandu, 2006). Regarding the accessibility and modifiability of individuals’ behaviors, a growing number of studies were conducted to underline the association of modifiable lifestyle factors with dementia (Baumgart *et al.*, 2015). For example, abuse of substances such as cigarettes (Ohara *et al.*, 2015) and alcohol (Ormstad *et al.*, 2016) and leisure activities such as TV watching (Shin *et al.*, 2013), reading (Lopes *et al.*, 2012), exercise (Wong *et al.*, 2016), and social activity (Wong *et al.*, 2016) have been studied substantially for their associations with dementia. However, little is known about their associations with each specific domain of cognitive function. In particular, smartphones, a very popular device currently, had been reported to have an independent facilitating effect on global cognition among old people (Ng *et al.*, 2012); hence, we included it and sought to detect its associations with different cognitive domains.

In this study, we aimed to understand the role of lifestyle factors – substance abuse (cigarettes and alcohol) and leisure activity (TV watching, reading, smartphone use, exercise, and social activity) – in cognitive health, particularly in different domains of cognitive function, and to guide individual interventions for specific kinds of decline in cognitive domains.

## Methods

### Participants

A cross-sectional study was conducted with individuals who were registered residents aged over 60 years from July 1st to October 20th in 2016. To create a baseline database, we proposed to cover 1% of overall registered individuals aged over 60 years in Xiamen, where there were 275.8 thousand eligible participants at the time of this survey. Hence, the calculated sample size was 2,758 individuals. Given a non-response rate of 15%, the sample size was inflated to 3,172.

A multistage random sampling method was used. The first stage of sampling involved all six districts in Xiamen, including two urban and four rural districts. Half of the sub-districts were randomly selected from each district in the second stage, and 44 communities in total were randomly chosen in the third stage of sampling. In the fourth stage, individuals were randomly selected in each community by controlling for gender and age, and the sampling size was determined according to its proportion of eligible older adults. Participants were excluded if they met any of the following criteria: (1) refused to participate; (2) were deaf, dumb, blind, or had any other physical disability that may hinder completion of the survey; or (3) had any major psychiatric illness or history of mental disorders. A total of 3,230 respondents were interviewed, of whom 3,061 (94.77%) were provided valid responses. Ethical review of this study was approved by the Committee of the School of Public Health, Xiamen University. Informed consent was obtained from each participant on the first page of the questionnaire.

### Measures

Cognitive function was measured by a brief and widely used cognitive screening tool – the Montreal Cognitive Assessment (MoCA), which has high sensitivity and specificity for detecting mild cognitive impairment (Nasreddine *et al.*, 2005). In this study, we used the MoCA-BJ, a Chinese version of the MoCA tailored to the local context, which has been reported to be a reliable and stable cognitive screening tool among Chinese older adults (Chen *et al.*, 2015). The MoCA-BJ assessed six cognitive domains: (1) the short-term memory recall task (five points) involved two learning trials of five nouns and delayed recall after approximately 5 minutes; (2) visuospatial abilities (four points) were assessed using the *Clock Drawing Test* (three points) and *Copy of the Cube* (one point); (3) multiple aspects of executive function (four points) were assessed using the *Modified Trail Making Test* (one point), *Animals Fluency* (one point), and a two-item verbal abstraction task (one point each); (4) attention (six points) was evaluated using a sustained attention task (*Number 1 Tapping Test*; 1 point), *Serial 7 Subtractions* (three points), *Digit Span Forward* (one point), and *Digit span backward* (one point); (5) language (six points) involved a three-item confrontation naming task with low-familiarity animals (lion, camel, and rhinoceros; three points), *Sentence Repetition* (two points), and the aforementioned fluency task (one point); and (6) orientation (six points) to time and place was evaluated (Nasreddine *et al.*, 2005). Higher scores indicate better cognitive functions. The details of the scale, including the contents and scoring rules, can be found at http://www.mocatest.org/. Of note, to reduce the educational bias of the MoCA, two points were added for old adults with ≤6 years of education, while one point was added for those with 7~12 years of education (the points were not added if the total score exceeded 30 points after the addition) (Tan *et al.*, 2015). Furthermore, participants whose original MoCA scores were less than cut-off points for dementia were excluded, these cut-off points were 11, 14, and 16 for ≤5, 6~8, and ≥9 years of education, respectively (Yin *et al.*, 2014). In the end, 2,617 participants were included in the following analyses. Since no valid local studies to date have suggested the educational adjustments for MoCA sub-domains, we then further divided our eligible respondents into three groups by their education level (≤5, 6~8, and ≥9 years of education).

Among the exposure factors of interest (lifestyle factors), participants were asked to choose the best option that represented their past experience of such behaviors. For substance abuse, there were three options for cigarette smoking and alcohol drinking: “never,” “smoking/drinking now,” and “have quit now.” For leisure activity, continuous numeric values were used for TV watching and reading, which were assessed by the questions “How many hours did you spend watching TV per day last month on average?” and “How many hours did you spend reading books or newspapers per week last month on average?,” respectively. Smartphone use and social activity were measured by answers to the questions, “Did you frequently use a smartphone last month?” and “Did you frequently engage in social activity last month?,” respectively, with two alternative options of “yes” or “no.” Exercise was assessed by asking “How many days on average did you exercise in one week?” with five options: “never,” “1~2 days,” “3~4 days,” “5~6 days,” and “every day.”

Additionally, some demographic characteristics (age, gender, area, education, and marital status) and some medical and health factors (body mass index (BMI), hypertension, diabetes, and depression) were considered as covariates. Among them, BMI was classified into four categories based on recommendations by the Working Group On Obesity in China of the Chinese Ministry of Health (Zhou, 2002): <18.5 kg/m^2^ for underweight, 18.5~23.9 kg/m^2^ for normal weight, 24~27.9 kg/m^2^ for overweight, and ≥28 kg/m^2^ for obese. Chronic diseases were assessed by asking “Do you suffer from the following physician-diagnosed chronic diseases (check all that apply)?” Hypertension and diabetes were two options among the listed chronic diseases. Finally, the 15-item Geriatric Depression Scale (GDS-15) was used, and those who scored more than 4 would then be deemed to have depression (Kurlowicz and Greenberg, 2007).

### Data analysis

First, we summarized the characteristics of participants by using descriptive statistics (proportion, mean, and standard deviations) for each cognitive domain and compared the mean difference of scores by using analysis of variance (ANOVA). Serial proportional odds models were used separately in three education-specific groups – MoCA scores in each cognitive domain as the ordinal dependent variable and lifestyle factors and other covariates as independent variables – to estimate the associations between lifestyle factors and specific cognitive functions. Taking the scores in executive ability as the ordinal dependent variable as an example, the proportional odds model estimates four logits (log odds) as follows:

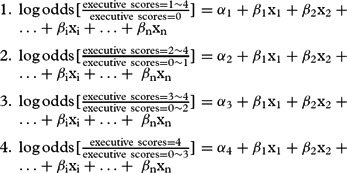

The model estimated separate intercepts for each logit (α_1_, α_2_, α_3_, and α_4_), but the proportional odds assumption limited the relationship between each explanatory variable and the dependent variable to be constant; that is, *β*_i_ in each logit was the same. Similarly, a batch of proportional odds models estimated 5, 4, 6, 6, and 6 logits when we used the MoCA scores in memory, visuospatial abilities, attention, language, and orientation as dependent variables, respectively. We also used similar models to test the relationship between lifestyle factors and general cognition by using MoCA-adjusted total score percentiles (P25, P50, and P75) as the ordinal dependent variable. Socio-demographic factors were controlled in all models aforementioned. The models were fit with maximum likelihood estimation using SAS version 9.4 (SAS Institute, Cary NC, 2017).

## Results

### Descriptive analyses

Among the 2,617 eligible participants, the overall mean age was 69.06 ± 7.06 years, with ages ranging from 60 to 96 years. Additionally, 54.03% of the participants were men and 54.41% were from urban areas. Nearly two-thirds attained less than nine years of education and 25.34% were unmarried. Regarding lifestyle factors, current smokers and drinkers accounted for 30.89% and 16.50%, respectively. Most of the respondents (94.72%) watched TV, but comparatively few participants (41.56%) read books or newspapers. Smartphone users accounted for over a quarter. Over half of the participants did not take part in social activity frequently, while one-third did not exercise for a single day per week or were overweight or obese. Participants suffering from hypertension, diabetes, or depression comprised 35.04%, 10.59%, and 9.81% of the sample, respectively. More detailed descriptive statistics are displayed in Table 1.

**Table 1.** Characteristics of 2,617 participants according to the MoCA scores and ANOVA results

			sub-score of moca in six cognitive domains (mean ± sd)					
	*N*(%)	moca score*	memory	visuospatial	executive	attention	language	orientation
Overall		21.97 ± 4.61	2.66 ± 1.88	2.06 ± 1.47	1.88 ± 1.14	4.98 ± 1.28	4.47 ± 1.37	5.72 ± 0.66
Age (years)		**<0.001**	**<0.001**	**<0.001**	**<0.001**	**<0.001**	**<0.001**	**0.001**
60~69	1,570(59.99)	22.68 ± 4.39	2.86 ± 1.82	2.21 ± 1.41	1.95 ± 1.14	5.17 ± 1.14	4.60 ± 1.35	5.75 ± 0.64
70~79	781(29.84)	21.58 ± 4.70	2.46 ± 1.94	2.08 ± 1.50	1.91 ± 1.13	4.88 ± 1.33	4.40 ± 1.38	5.69 ± 0.67
>=80	266(10.16)	19.03 ± 4.25	2.12 ± 1.84	1.17 ± 1.41	1.42 ± 1.01	4.19 ± 1.54	3.96 ± 1.40	5.60 ± 0.75
Gender		**<0.001**	0.051	**<0.001**	**<0.001**	**<0.001**	**<0.001**	0.118
Male	1,414(54.03)	22.78 ± 4.31	2.59 ± 1.88	2.43 ± 1.38	2.06 ± 1.14	5.20 ± 1.11	4.69 ± 1.27	5.74 ± 0.67
Female	1,203(45.97)	21.03 ± 4.76	2.75 ± 1.87	1.63 ± 1.44	1.66 ± 1.10	4.72 ± 1.41	4.22 ± 1.44	5.69 ± 0.66
Area		**<0.001**	**<0.001**	**<0.001**	**<0.001**	**<0.001**	**<0.001**	**<0.001**
Rural	1,193(45.59)	20.78 ± 4.46	2.26 ± 1.96	1.84 ± 1.46	1.67 ± 1.11	4.82 ± 1.30	4.18 ± 1.39	5.64 ± 0.77
Urban	1,424(54.41)	22.98 ± 4.49	3.00 ± 1.74	2.25 ± 1.44	2.05 ± 1.14	5.11 ± 1.24	4.71 ± 1.31	5.77 ± 0.56
Education (years)		**<0.001**	**<0.001**	**<0.001**	**<0.001**	**<0.001**	**<0.001**	**<0.001**
<=5	1,124(43.00)	19.32 ± 4.33	2.18 ± 1.92	1.23 ± 1.30	1.35 ± 0.97	4.34 ± 1.45	3.88 ± 1.41	5.58 ± 0.80
6~8	599(22.92)	22.93 ± 3.81	2.80 ± 1.86	2.25 ± 1.32	1.85 ± 1.02	5.28 ± 0.98	4.54 ± 1.25	5.78 ± 0.60
>=9	891(34.09)	24.69 ± 3.46	3.13 ± 1.71	2.96 ± 1.15	2.54 ± 1.05	5.58 ± 0.76	5.18 ± 1.01	5.85 ± 0.46
Marital status		**<0.001**	**0.009**	**<0.001**	**<0.001**	**<0.001**	**<0.001**	**<0.001**
Unmarried	631(25.33)	20.38 ± 4.54	2.47 ± 1.92	1.50 ± 1.43	1.56 ± 1.03	4.65 ± 1.43	4.14 ± 1.43	5.62 ± 0.75
Married	1,860(74.67)	22.57 ± 4.49	2.71 ± 1.85	2.26 ± 1.43	1.99 ± 1.15	5.10 ± 1.19	4.59 ± 1.34	5.74 ± 0.64
Cigarette		**<0.001**	**0.033**	**<0.001**	**0.004**	**<0.001**	**<0.001**	0.053
Never smoke	1,606(62.18)	21.67 ± 4.72	2.74 ± 1.85	1.92 ± 1.50	1.82 ± 1.14	4.87 ± 1.34	4.37 ± 1.41	5.73 ± 0.62
Previous smoke	179(6.93)	22.78 ± 4.13	2.56 ± 1.82	2.38 ± 1.33	2.09 ± 1.14	5.25 ± 1.06	4.74 ± 1.20	5.80 ± 0.51
Current smoke	798(30.89)	22.43 ± 4.44	2.53 ± 1.93	2.27 ± 1.39	1.93 ± 1.12	5.14 ± 1.17	4.61 ± 1.31	5.68 ± 0.77
Alcohol		**<0.001**	0.829	**<0.001**	**<0.001**	**<0.001**	**<0.001**	0.066
Never drink	2,059(79.38)	21.74 ± 4.64	2.66 ± 1.88	1.98 ± 1.48	1.82 ± 1.13	4.93 ± 1.30	4.41 ± 1.39	5.71 ± 0.66
Previous drink	107(4.12)	22.43 ± 4.58	2.57 ± 1.88	2.36 ± 1.27	2.08 ± 1.20	4.91 ± 1.27	4.51 ± 1.22	5.62 ± 0.82
Current drink	428(16.50)	23.07 ± 4.33	2.70 ± 1.89	2.45 ± 1.36	2.09 ± 1.14	5.22 ± 1.14	4.78 ± 1.28	5.78 ± 0.61
TV watching/hours per day		**<0.001**	0.225	**0.033**	0.054	**<0.001**	**<0.001**	**0.004**
0	133(5.28)	20.59 ± 5.24	2.38 ± 1.85	1.91 ± 1.56	1.64 ± 1.20	4.63 ± 1.53	3.95 ± 1.6	5.53 ± 0.83
0.1~2	1,072(42.52)	21.93 ± 4.70	2.68 ± 1.87	2.12 ± 1.48	1.89 ± 1.16	4.95 ± 1.28	4.37 ± 1.37	5.70 ± 0.68
2.1~4	937(37.17)	22.40 ± 4.52	2.74 ± 1.86	2.11 ± 1.45	1.93 ± 1.11	5.10 ± 1.22	4.64 ± 1.34	5.74 ± 0.66
>4	379(15.03)	21.68 ± 4.27	2.62 ± 1.92	1.89 ± 1.43	1.84 ± 1.09	4.91 ± 1.25	4.50 ± 1.32	5.76 ± 0.58
Reading/hours per week		**<0.001**	**<0.001**	**<0.001**	**<0.001**	**<0.001**	**<0.001**	**<0.001**
0	1,495(58.44)	20.52 ± 4.55	2.47 ± 1.9	1.60 ± 1.42	1.53 ± 1.03	4.66 ± 1.41	4.09 ± 1.40	5.63 ± 0.78
0.1~3.5	577(22.56)	24.05 ± 3.89	2.99 ± 1.84	2.65 ± 1.30	2.33 ± 1.11	5.42 ± 0.90	5.09 ± 1.11	5.85 ± 0.44
>3.5	486(19.00)	24.13 ± 3.76	2.88 ± 1.76	2.81 ± 1.21	2.41 ± 1.10	5.45 ± 0.86	4.97 ± 1.13	5.83 ± 0.43
Smartphone use		**<0.001**	**<0.001**	**<0.001**	**<0.001**	**<0.001**	**<0.001**	**<0.001**
No	1,821(70.04)	20.99 ± 4.55	2.47 ± 1.91	1.77 ± 1.46	1.66 ± 1.08	4.78 ± 1.36	4.24 ± 1.40	5.67 ± 0.72
Yes	779(29.96)	24.32 ± 3.83	3.11 ± 1.72	2.74 ± 1.24	2.39 ± 1.11	5.44 ± 0.90	5.01 ± 1.15	5.83 ± 0.51
Social activity		**<0.001**	**<0.001**	**<0.001**	**<0.001**	**<0.001**	**<0.001**	**<0.001**
No	1,421(55.16)	21.31 ± 4.57	2.45 ± 1.89	1.89 ± 1.49	1.76 ± 1.12	4.87 ± 1.32	4.31 ± 1.41	5.67 ± 0.71
Yes	1,155(44.84)	22.85 ± 4.51	2.93 ± 1.82	2.29 ± 1.41	2.04 ± 1.14	5.13 ± 1.20	4.68 ± 1.30	5.77 ± 0.60
Exercise/days per week		**<0.001**	**<0.001**	**<0.001**	**<0.001**	**<0.001**	**<0.001**	**<0.001**
0	836(32.85)	20.89 ± 4.45	2.36 ± 1.93	1.77 ± 1.45	1.65 ± 1.08	4.77 ± 1.36	4.18 ± 1.39	5.61 ± 0.80
1~4	428(16.82)	21.88 ± 4.67	2.64 ± 1.87	2.10 ± 1.46	1.87 ± 1.13	4.99 ± 1.24	4.51 ± 1.35	5.75 ± 0.57
5~7	1,281(50.33)	22.77 ± 4.53	2.91 ± 1.81	2.25 ± 1.45	2.03 ± 1.15	5.12 ± 1.21	4.66 ± 1.34	5.78 ± 0.58
BMI (kg/m^2^)		**<0.001**	0.113	**<0.001**	**0.011**	**0.002**	**0.039**	0.894
<18.5	203(8.16)	20.79 ± 4.38	2.42 ± 1.98	1.74 ± 1.53	1.65 ± 1.10	4.70 ± 1.38	4.26 ± 1.41	5.70 ± 0.67
18.5~23.9	1,457(58.58)	22.11 ± 4.62	2.68 ± 1.88	2.14 ± 1.44	1.89 ± 1.12	5.02 ± 1.24	4.47 ± 1.37	5.72 ± 0.69
24.0~27.9	680(27.34)	22.37 ± 4.57	2.81 ± 1.80	2.15 ± 1.45	1.94 ± 1.18	5.07 ± 1.25	4.56 ± 1.37	5.74 ± 0.60
>=28	147(5.91)	21.58 ± 4.38	2.65 ± 1.74	1.73 ± 1.49	1.78 ± 1.13	4.89 ± 1.23	4.57 ± 1.38	5.71 ± 0.68
Hypertension		0.476	0.731	0.849	0.022	0.868	0.092	0.087
No	1,676(64.96)	21.94 ± 4.58	2.66 ± 1.87	2.06 ± 1.46	1.84 ± 1.14	4.98 ± 1.27	4.44 ± 1.37	5.71 ± 0.69
Yes	904(35.04)	22.07 ± 4.65	2.69 ± 1.88	2.05 ± 1.47	1.95 ± 1.14	4.97 ± 1.29	4.54 ± 1.37	5.75 ± 0.59
Diabetes		0.751	0.671	0.743	0.178	0.726	0.051	0.736
No	2,288(89.41)	21.97 ± 4.62	2.67 ± 1.88	2.06 ± 1.46	1.87 ± 1.14	4.98 ± 1.28	4.45 ± 1.38	5.72 ± 0.66
Yes	271(10.59)	22.06 ± 4.57	2.62 ± 1.81	2.03 ± 1.51	1.97 ± 1.14	4.95 ± 1.30	4.63 ± 1.30	5.74 ± 0.62
Depression		**<0.001**	**<0.001**	**<0.001**	**<0.001**	**<0.001**	**<0.001**	**<0.001**
No	2,290(90.19)	22.26 ± 4.56	2.74 ± 1.87	2.12 ± 1.45	1.91 ± 1.15	5.03 ± 1.24	4.53 ± 1.36	5.74 ± 0.62
Yes	249(9.81)	19.74 ± 4.43	1.94 ± 1.81	1.65 ± 1.47	1.60 ± 1.07	4.51 ± 1.47	4.08 ± 1.40	5.49 ± 0.94

*Note:* MoCA Score* has been adjusted according to the education year. Significant p-values (p < 0.05) are marked in bold.

Overall, the education-adjusted mean MoCA score was 21.97 ± 4.61. Participants who attained higher scores in general cognition and each cognitive domain were more likely to be younger, male, married, living in urban areas, and more formally educated. The ANOVA indicated that the mean scores of each cognitive domain were significantly different in almost all groups with lifestyle factors. Higher scores were more likely for ex-smokers or current drinkers. Participants who watched TV, read books/newspapers, used smartphones, exercised, or engaged in social activity scored higher than those who did not. Participants with hypertension or diabetes had similar scores as those without, while elderly individuals with depression showed significantly lower scores than those without depression in all six cognitive domains.

### Odds ratios of lifestyle factors for better cognitive function

**Figure 1.**
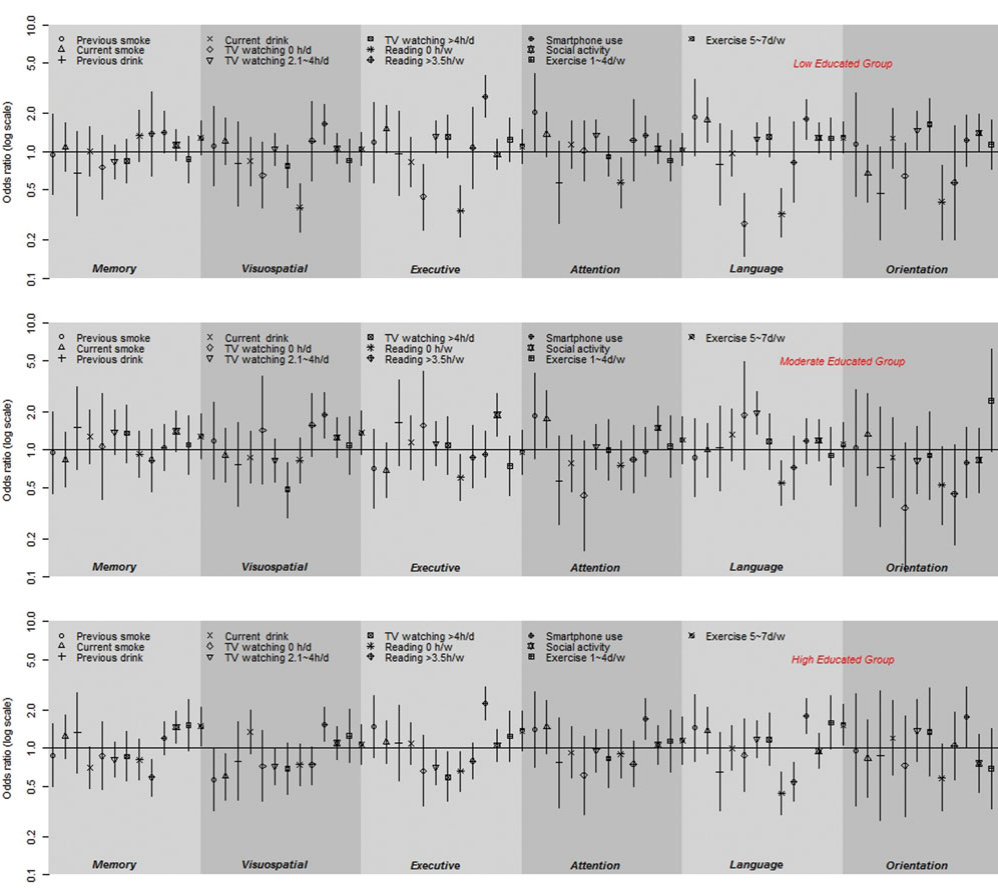
Odds ratios and 95% confidence intervals for better cognitive functions in six domains among three education-specific groups. Proportional odds models were adjusted for background factors (age, gender, area, education, marital status), and medical and health factors (BMI, hypertension, diabetes, depression) in all models. Symbols represent the point estimates (ORs) while vertical bars around the symbols are the corresponding 95% CIs. Reference groups: Cigarette: never smoke; Alcohol: never drink; TV watching: 0.1~2h/d; Reading: 0.1~3.5h/w; Smartphone use: no; Social activity: no; Exercise: 0d/w.

A series of proportional odds models predicting better cognitive function were fitted for the general MoCA (Table 2) and six sub-domains of MoCA (Figure 1) in the three education-specific groups. For each model, a *χ*^2^ score test supported the appropriateness of the proportional odds model.

**Table 2.** The odds ratios (95% confidential interval) of lifestyle characteristics for higher MoCA scores in three education-specific groups

	moca scores			
lifestyle-related		education	education	education
factors	overall	year <=5	year 6~8	year >=9
Substance abuse				
Cigarette (ref = never smoke)				
Previous smoke	1.27(0.87 – 1.85)	**2.22(1.07 – 4.61)**	0.99(0.49 – 1.99)	1.21(0.69 – 2.11)
Current smoke	1.07(0.83 – 1.36)	1.35(0.90 – 2.04)	0.95(0.59 – 1.54)	1.18(0.80 – 1.75)
Alcohol (ref = never drink)				
Previous drink	0.88(0.57 – 1.34)	0.69(0.32 – 1.49)	1.61(0.75 – 3.43)	0.80(0.40 – 1.58)
Current drink	1.13(0.88 – 1.44)	1.22(0.79 – 1.88)	0.98(0.62 – 1.55)	1.00(0.69 – 1.45)
Leisure activity				
TV watching/hours per day (ref = 0.1~2)				
0	0.69(0.46 – 1.02)	**0.48(0.28 – 0.85)**	1.54(0.58 – 4.10)	0.63(0.34 – 1.19)
2.1~4	1.07(0.89 – 1.29)	1.25(0.93 – 1.66)	1.35(0.92 – 1.97)	0.80(0.59 – 1.09)
>4	0.88(0.68 – 1.12)	1.18(0.82 – 1.71)	0.88(0.54 – 1.45)	0.77(0.49 – 1.20)
Reading/hours per week (ref = 0.1~3.5)				
0	**0.49(0.39 – 0.62)**	**0.38(0.24 – 0.61)**	**0.66(0.44 – 0.98)**	**0.59(0.41 – 0.85)**
>3.5	**0.69(0.53 – 0.90)**	1.25(0.56 – 2.79)	0.94(0.54 – 1.63)	**0.53(0.38 – 0.73)**
Smartphone use (ref = no)	**1.71(1.40 – 2.09)**	**1.86(1.28 – 2.69)**	1.21(0.81 – 1.81)	**2.01(1.49 – 2.70)**
Social activity (ref = no)	**1.27(1.07 – 1.51)**	1.10(0.84 – 1.44)	1.42(0.99 – 2.03)	1.30(0.97 – 1.73)
Exercise/days per week (ref = 0)				
1~4	0.89(0.69 – 1.15)	0.76(0.53 – 1.11)	0.90(0.54 – 1.51)	1.33(0.85 – 2.11)
5~7	1.10(0.91 – 1.34)	1.07(0.80 – 1.43)	1.00(0.68 – 1.48)	**1.48(1.04 – 2.10)**

*Note:* Background factors (age, gender, area, education, and marital status) and medical and health factors (BMI, hypertension, diabetes, and depression) were controlled in all models. Significant p-values (p < 0.05) are marked in bold.

As Table 2 shows, previous or current smoking and drinking was not associated with MoCA scores in the general population, but unexpectedly, ex-smokers in the low education group demonstrated better cognition (OR = 2.22) than never smokers. Reading, smartphone use (OR = 1.71), and engagement in social activity (OR = 1.27) all contributed to better cognition. TV watching and exercise were not associated with general cognition in the whole population; however, for the low education group, never watching TV was harmful (OR = 0.48), and for the high educated group, exercising 5~7 days per week was beneficial (OR = 1.48).

Figure 1 presents the odds ratios and 95% confidence intervals for better cognitive function in the six domains among the three education-specific groups. Compared with never smokers, ex-smokers, and current smokers performed better in attention (OR(95%CI): 2.05(1.02 – 4.14)) and language (OR(95%CI): 1.78 (1.18 – 2.67)), respectively, in the low education group, while in the high education group, current smokers performed worse in visuospatial function (OR(95%CI): 0.60 (0.39 – 0.91)). Drinking showed no association with any sub-domain of cognition in all three education-specific groups. For old adults in the low education group, compared to watching TV less than 2 hours, never watching was detrimental to executive ability (OR(95%CI): 0.44(0.24 – 0.79)) and language (OR(95%CI): 0.27(0.15 – 0.47)), and longer watching times were beneficial to attention (2.1~4 hours/day: OR(95%CI): 1.35(1.02 – 1.79)) and orientation (2.1~4 hours/day: OR(95%CI): 1.47 (1.03 – 2.08); >4 hours/day: OR(95%CI): 1.63(1.02 – 2.6)). However, excessive TV watching (>4 hours/day) was inversely associated with visuospatial function (OR(95%CI): 0.49 (0.29 – 0.8)) for the moderately educated elderly, even though modest TV watching (2~4 hours/day) was conversely related to visuospatial function (OR(95%CI): 0.72(0.52 – 0.99)) and executive ability (OR(95%CI): 0.71(0.52 – 0.97)) for the elderly with a high education level. Similarly, compared to reading no more than 3.5 hours per week, never reading was harmful to executive ability and language in all three education-specific groups, and for the low education group in particular, never reading showed an inverse association with multi-domain cognitive functions involving visuospatial ability (OR(95%CI): 0.36(0.23 – 0.56)), executive ability (OR(95%CI): 0.34(0.21 – 0.54)), attention (OR(95%CI): 0.57(0.36 – 0.89)), language (OR(95%CI): 0.32 (0.21 – 0.51)), and orientation (OR(95%CI): 0.40 (0.2 – 0.78)). However, comparatively longer reading (>3.5 hours/week) was detrimental to memory (OR(95%CI): 0.59(0.42 – 0.82)) and language (OR(95%CI): 0.54(0.38 – 0.77)) in the high education group. Smartphone users had the edge over non-smartphone users in five cognitive domains for the high education group, including visuospatial ability (OR(95%CI): 1.54(1.14 – 2.1)), executive ability (OR(95%CI): 2.25(1.67 – 3.03)), attention (OR(95%CI): 1.71(1.18 – 2.46)), language (OR(95%CI): 1.80(1.31 – 2.47)), and orientation (OR(95%CI): 1.77(1.02 – 3.04)), while this edge was limited within comparatively fewer sub-domains of cognition for the low and moderate education groups. Those frequently engaged in social activity also performed better in multi-domain cognitive functions for all the groups, though this beneficial effect was significant only for executive ability (OR(95%CI): 1.88(1.29 – 2.75)) within old adults in the moderate education group and for memory (OR(95%CI): 1.47(1.09 – 1.97)) within the high education group. Likewise, those who exercised more than five days a week showed superior results to those who did not exercise for a single day per week in almost all the sub-domains of cognition for all the groups, but the findings were significantly better only in memory (OR(95%CI): 1.49(1.05 – 2.12)) and language (OR(95%CI): 1.53(1.06 – 2.21)) for old adults in the high education group.

## Discussion

Based on our large-scale survey data, we tried to detect the associations of modifiable lifestyle factors with multi-domain cognitive functions in three education-specific groups. Leisure activity was associated with multi-domain cognition, while substance abuse showed only a limited association. It is worth noting that for the elderly with a low education level, leisure activities such as TV watching, reading, and smartphone use effectively contributed to better cognition, while reading and exercise were beneficial to cognition for old adults with a moderate and high education, respectively.

### Substance abuse, on the whole, showed a limited association with cognition

The substances considered in this study were cigarettes and alcohol. Our study indicated that smoking was not associated with cognition, but previous smoking showed a positive relationship among elderly participants in the low education group; more specifically, previous and current smoking might be beneficial to attention and language, respectively, for people with a moderate or low education level, while current smoking might be detrimental to visuospatial functions for old adults with a high education level. Previous experimental findings also found that smoking could enhance attention and that this enhancement may be induced by nicotine (Amitai and Markou, 2008; Rezvani *et al.*, 2008). Furthermore, Daniela and colleagues found that smokers performed better in verbal memory and language fluency than non-smokers (Caldirola *et al.*, 2013), and this result might be due to social smoking (Schane *et al.*, 2009), hence stimulating language ability. However, thus far, studies about the association between cigarettes and cognition have not reached a consensus. A cohort study in Britain assessed the association between smoking and cognition by gender and found that male smokers, compared to never smokers, experienced faster cognitive decline in global cognition and executive function, whereas no difference appeared in women as smoking status varied (Sabia *et al.*, 2012). Considering the controversy over its benefits for cognition and the confirmation of its detrimental effect on pulmonary diseases and cardiovascular diseases, we call on the public not to smoke.

Drinking showed no association with general cognition or any sub-domain of cognition in our study. Drinking has been found to be similarly irrelevant to general cognition, verbal memory, semantic fluency, and attention in a cohort study, but the heaviest drinkers had the lowest phonemic fluency scores (Gross *et al.*, 2011). However, Tiia's findings suggested frequent and infrequent drinkers had better executive function and episodic memory than never drinkers (Ngandu, 2006). Dose-effect and beverage type might, to some extent, elucidate this discrepancy. A growing body of reports showed a U-shaped relationship, i.e. a beneficial effect on cognition related to moderate alcohol drinking (Reas *et al.*, 2016; Shimizu *et al.*, 2016). Moreover, several studies showed that only wine had a protective effect on cognition (Gu *et al.*, 2014). In this study, however, we did not study the amount of alcohol consumed or the type of drink. We recommend that future studies explore the association of alcohol consumption and types of alcohol with domain-specific cognitive function.

### Leisure activity associated with multi-domain cognitive functions

For the elderly in the low education group, TV watching, smartphone use, and reading enhanced multiple domains of cognition. It was reported that TV watching, smartphone use, and reading generally could be regarded as cognitive activities, which are good for cognition (Doi *et al.*, 2013). Specifically, TV can serve as a source of media that equips the elderly with the latest information (Ostlund, 2010), which might help them achieve better temporal orientation. Furthermore, the information on TV attracts viewers largely with sounds and colorful images (Sigman, 2007), and this, to some extent, might enhance their attention. Additionally, watching TV might increase the communication between TV viewers and others, as they can share and discuss conversation topics from TV watching (Sun *et al.*, 2016). Similar benefits from reading might be akin to TV watching, as mentioned above. In addition, smartphone users generally have to be familiar with the spatial layout of the number pad and various functional buttons. Additionally, they might master how to use some complicated applications such as the camera, video, and social media (Ballagas *et al.*, 2006), and all of this therefore could serve as a form of cognitive training that enhances multi-domain cognitive ability.

A less sedentary lifestyle was beneficial for the highly educated old adults. Moderate (2.1~4 hours/day) or excessive (>4 hours/day) TV watching (though not significantly) and comparatively longer reading (>3.5 hours/week) in this study showed negative associations with cognition, while adequate exercise (5~7 days/week) showed a positive relationship in the highly educated old adults. A previous study also found that excessive TV watching might negatively affect cognition (Hoang *et al.*, 2016) because too much time spent watching TV generally meant excessive sedentary behavior and less physical activity, which was not good for cognition (Falck *et al.*, 2016). Similar explanations were also applicable to reading. A cohort study conducted with older Mexican Americans also indicated that greater exercise was associated with reduced memory function decline (Ottenbacher *et al.*, 2014) and better language (Salinas *et al.*, 2014).

### Memory with comparatively less positive factors

Among the six sub-domains, the most affected by age was memory (Riddle, 2007). It should be noted that fewer factors observed in the current study were significantly associated with memory than with attention. In addition, those cognitive enhancement factors affecting memory, including social activity and exercise, worked only for the high education group. For one, this might be because memory was a complex process involving encoding, storage, and retrieval. In addition, the information gained from TV watching or reading was more likely to be at the input stage, which meant that the information input may not be encoded or stored well, let alone be retrieved. In contrast, regarding more social activity, such as staying in contact with friends or playing mahjong (in reality or by using a mobile app), as the output, this activity forced the elderly to use information or skills they had learned before, which required an intact memory process of encoding, storage, and retrieval. For another, by using molecular systems associated with synaptic plasticity and energy metabolism, exercise can activate the neural circuitry, which is important for learning (Gomez-Pinilla and Hillman, 2013). Given that memory decline was one of the most common complaints among older adults, we appeal to the public to live an active lifestyle and especially to engage in more social activity and exercise.

Of note, our ANOVA results indicated that participants with hypertension or diabetes had similar MoCA scores as those without. To date, studies of hypertension and diabetes have not indicated a consistent relationship with cognition. A meta-analysis even found that hypertension treatments may reduce the risk of cognitive decline (Rouch *et al.*, 2015). In addition, a recent study indicated that patients with diabetes might have higher odds of having vascular dementia but lower odds of having Alzheimer's dementia (Sherzai *et al.*, 2016).

### Limitations

This study has a number of strengths. One of the main strengths is that we have taken into account a broad range of cognitive functions that had not been studied simultaneously before. Many previous studies focused mainly on general cognition or a fraction of cognitive domains. Moreover, we explored the associations between domain-specific cognitive function and modifiable lifestyle factors in education-specific groups, which offered a new perspective for specific individual interventions.

Nevertheless, some limitations should also be acknowledged. First, detailed information on the independent variables (for example, the type of drinking beverage and drinking frequency) was not collected, which impeded a deeper understanding of their effects on cognition. Second, domain-specific cognitive functions were assessed by sub-scores of the MoCA, which has been reported to be subject to educational bias. However, to reduce such bias, we divided our respondents into three groups according to participants’ education level, which was recommended by a similar local study – that study used the MoCA in a large sample of community-dwelling old adults whose dialects were also different from Mandarin to detect dementia and mild cognitive impairment and recommended optimal cut-offs stratified by education level.

## Conclusion

This study contributes to the literature as the first study to describe lifestyle factors in relation to multi-domain cognitive functions according to participants’ education levels. In conclusion, the present results suggested that for elderly with a low education level, leisure activities such as TV watching, smartphone use, and reading could enhance multiple domains of cognition; for old adults with a high education level, a less sedentary lifestyle involving engaging in more exercise, avoiding excessive TV watching and reading was beneficial; and for the moderately educated elderly, reading is a good way to improve general cognition, executive function, and language. Thus, individual intervention strategies should be designed according to specific educational groups and sub-domains. More detailed information on the effect of lifestyle on specific cognitive domains and longitudinal analyses will be needed in the future.

## Conflict of interest

None.

## Description of authors’ roles

Manqiong Yuan and Jia Chen participated equally in study conception, design, and analysis, interpretation of results, and drafting of the paper. Yaofeng Han, Xingliang Wei, Zirong Ye, and Liangwen Zhang contributed significant time and labor to the survey. Y. Alicia Hong assisted with interpretation of results and made critical revisions to the paper for important intellectual content. Ya Fang supervised the study, participated in study conception and design and interpretation of results, and made critical revisions to the paper for important intellectual content. All the authors approved the final version for submission.
